# Physicians’ Perceptions of Clinical Utility of a Digital Health Tool for Electronic Patient-Reported Outcome Monitoring in Real-Life Hematology Practice. Evidence From the GIMEMA-ALLIANCE Platform

**DOI:** 10.3389/fonc.2022.826040

**Published:** 2022-03-17

**Authors:** Fabio Efficace, Andrea Patriarca, Mario Luppi, Leonardo Potenza, Giovanni Caocci, Agostino Tafuri, Francesca Fazio, Claudio Cartoni, Maria Teresa Petrucci, Ida Carmosino, Riccardo Moia, Gloria Margiotta Casaluci, Paola Boggione, Elisabetta Colaci, Davide Giusti, Valeria Pioli, Francesco Sparano, Francesco Cottone, Paolo De Fabritiis, Nicolina Rita Ardu, Pasquale Niscola, Isabella Capodanno, Anna Paola Leporace, Sabrina Pelliccia, Elisabetta Lugli, Edoardo La Sala, Luigi Rigacci, Michelina Santopietro, Claudio Fozza, Sergio Siragusa, Massimo Breccia, Paola Fazi, Marco Vignetti

**Affiliations:** ^1^ Data Center and Health Outcomes Research Unit, Italian Group for Adult Hematologic Diseases (GIMEMA), Rome, Italy; ^2^ Division of Hematology, Department of Translational Medicine, University of Eastern Piedmont, Novara, Italy; ^3^ Hematology Unit and Chair, Azienda Ospedaliera Universitaria di Modena, Department of Medical and Surgical Sciences, University of Modena and Reggio Emilia, Modena, Italy; ^4^ Department of Medical Sciences and Public Health, Businco Hospital, University of Cagliari, Cagliari, Italy; ^5^ Azienda Ospedaliera Sant’Andrea, Rome, Italy; ^6^ Hematology, Department of Translational and Precision Medicine, Azienda Ospedaliera Policlinico Umberto I, Sapienza University of Rome, Rome, Italy; ^7^ Hematology Unit, Sant’Eugenio Hospital, Rome, Italy; ^8^ Hematology Unit, Azienda Unità Sanitaria Locale-IRCCS di Reggio Emilia, Reggio Emilia, Italy; ^9^ U.O. di Ematologia e Trapianti di cellule Staminali. A.O.S. S. Camillo-Forlanini, Rome, Italy; ^10^ Department of Medical, Surgical and Experimental Sciences, University of Sassari, Sassari, Italy; ^11^ Policlinico Paolo Giaccone, Unit of Haematology, Department of Health Promotion, Mother and Child Care, Internal Medicine and Medical Specialties (ProMISE), University of Palermo, Palermo, Italy

**Keywords:** digital health, symptoms, quality of life, hematology, patient-reported outcomes (PROs), leukemia, multiple myeloma, lymphoma

## Abstract

Digital health tools are increasingly being used in cancer care and may include electronic patient-reported outcome (ePRO) monitoring systems. We examined physicians’ perceptions of usability and clinical utility of a digital health tool (GIMEMA-ALLIANCE platform) for ePRO monitoring in the real-life practice of patients with hematologic malignancies. This tool allows for the collection and assessment of ePROs with real-time graphical presentation of results to medical staff. Based on a predefined algorithm, automated alerts are sent to medical staff. Participating hematologists completed an online survey on their experience with the platform. Of the 201 patients invited to participate between December 2020 and June 2021 (cut-off date for current analysis), 180 (90%) agreed to enter the platform and had a median age of 57 years. Twenty-three hematologists with a median age of 42 years and an average of 17 years of experience in clinical practice were surveyed. All hematologists agreed or strongly agreed that the platform was easy to use, and 87%, agreed or strongly agreed that ePROs data were useful to enhance communication with their patients. The majority of physicians (78%) accessed the platform at least once per month to consult the symptom and health status profile of their patients. The frequency of access was independent of physician sex (*p*=0.393) and years of experience in clinical practice (*p*=0.404). In conclusion, our preliminary results support the clinical utility, from the perspective of the treating hematologist, of integrating ePROs into the routine cancer care of patients with hematologic malignancies.

## Introduction

Patients with cancer typically experience disease- and treatment-related symptoms that affect their health-related quality of life (HRQoL). Therefore, it is critical to capture the patient experience *via* validated patient-reported outcome (PRO) measures that provide unique information, unobtainable by other sources of more traditional clinical and laboratory measures. For example, PROs, such as functional aspects or symptoms reported by patients themselves, provide independent prognostic information for survival (1, 2). Additionally, there is ample literature documenting that clinicians often underestimate the severity of their patients’ symptoms (3–6).

The assessment of PROs has been historically confined to clinical research settings; however, in recent years, we have seen a greater interest in using PROs in clinical practice in an effort to improve the quality of patient care. Indeed, systematic evaluation of PROs in routine practice has been found to be associated with several benefits, including improved symptom control, HRQoL, patient satisfaction, as well as improved physician-patient communication and decreased hospitalizations and emergency department visits (7–10).

The inclusion of PROs in routine practice settings has been facilitated by advances in digital health technology, which now allows the implementation of PROs into electronic formats that can be administered remotely *via* online platforms (11). Two recent randomized controlled trials (RCTs), including patients with several types of cancer during chemotherapy, showed that remote symptom monitoring with electronic PROs (ePROs) was associated with reduced symptom burden and improved HRQoL outcomes (12, 13). Remarkably, the systematic monitoring of PROs *via* web-based platforms has also been found to be associated with improved overall survival in patients with advanced cancers (14–17).

The recent coronavirus disease pandemic has further boosted the adoption of digital health tools that could facilitate remote patient monitoring during emergencies, making ePROs even more critical in enhancing patient-centered care. However, implementation of ePRO monitoring in the routine care of patients with hematologic malignancies has been less documented in the literature (18), and only recently have we seen valuable evidence in this area (19, 20). In any case, there is a paucity of information about users’ perceptions of the clinical utility of digital health tools in routine care.

Late in 2020, the Gruppo Italiano Malattie Ematologiche dell’Adulto (GIMEMA) developed a digital health tool for adult patients with hematological malignancies (GIMEMA-ALLIANCE platform) (21) with the main goal of facilitating patient-centered care in routine practice.

We herein report a survey conducted to better understand the hematologists’ perceptions of usability and clinical utility of this platform in real-life practice.

## Materials and Methods

### Study Design and Participants

Adult patients with a diagnosis of any hematologic malignancies according to the 2016 World Health Organization classification (22), who signed a written informed consent form, were eligible for enrollment in the GIMEMA-ALLIANCE platform. For the purpose of this project, patients could be included regardless of their type of therapy or individual characteristics, including age, level of education, or presence of comorbidities. After registration, patients were given (by their treating hematologist) a personal password to access the patient portal and complete a PRO survey that assessed aspects related to HRQoL, symptoms, and medication adherence. PRO measures include the European Organization for Research and Treatment of Cancer Quality of Life Questionnaire–Core (EORTC QLQ-C30) (23), four items from the EORTC Item Library (24), and the shortened 7-item Adherence to Refills and Medications Scale (ARMS-7) (25). These measures were selected based on their clinical relevance for the population under consideration. Indeed, the PRO questionnaires and items included in the platform, cover several aspects which are of importance across various hematological malignancies and have been widely used in previous studies. Each patient entering the platform has to be followed up for two years from the date of registration. As of January 2022, the platform includes 420 patients with hematologic malignancies, and 23 centers have obtained ethical approval to participate to this study. PRO results are available for both patients and physicians and are displayed graphically (in real time) with colored bars indicating the presence or absence of a clinically important problem or symptom. An example of the interfaces of the platform with the clinician with regard to display of functional aspects and symptoms is reported in the Appendix (**Supplementary Figures 1, 2**). Treating hematologists were required to collect clinical and socio-demographic information at baseline (e.g., patients’ and physicians’ characteristics, disease status at study entry) and every 3 months at follow-up (e.g., disease progression and survival status). Given the real-life nature of this study, no specific time-points were preplanned for the completion of the PRO survey. However, the platform is currently designed to send automated reminders to patients for completing the Survey after one week from registration (if this has not been completed within the first week from study inclusion), and thereafter every two weeks from the first PRO survey completion. In addition, physicians are encouraged (by the GIMEMA-ALLIANCE management team) to emphasize to their patients the importance of possibly completing the survey on a regular basis and, in any case, just few days before a planned clinical visit. The rationale for this latter aspect is that of providing a basis (updated information on patient’s HRQoL and symptoms) for further discussion during the clinical consultation. This study was registered at ClinicalTrials.gov (NCT04581187).

### Overview of the GIMEMA-ALLIANCE Infrastructure

The GIMEMA-ALLIANCE platform is hosted in the Computer-based Health Evaluation System (CHES) infrastructure, a software used worldwide for the electronic collection, analysis, and presentation of ePROs (26). Full details of the development process and architecture of the GIMEMA-ALLIANCE platform, including the study rationale and the implementation of ePRO measures, as well as clinical data collected, have been described previously (21). Briefly, the platform consists of two dedicated secure portals, the patient (https://alliance.gimema.it) and physician (https://physician-alliance.gimema.it) portals. Based on a predefined algorithm, the treating hematologists and medical staff receive automated email alerts following the presence of clinically important problems, symptoms, or problems with adherence to therapy. The definition of clinically important problems and symptoms is based on previously defined evidence-based thresholds for the EORTC QLQ-C30 (27). Once the alert is received, and depending on the types and frequencies of the alerts received, the physician may decide to contact the patient by phone, schedule a face-to-face visit, or arrange a video-consultation within the GIMEMA-ALLIANCE platform. Indeed, the possibility of video consultations is an additional feature of this tool. A specific standard operating procedure (SOP) on “how to handle e-mail alerts” was not developed because the platform is open to patients with any hematologic malignancy, hence representing a wide range of patients with different clinical conditions and different needs. Therefore, the protocol stipulated that physicians are free to decide which action they feel most appropriate for their specific patients.

**Figure 1:**
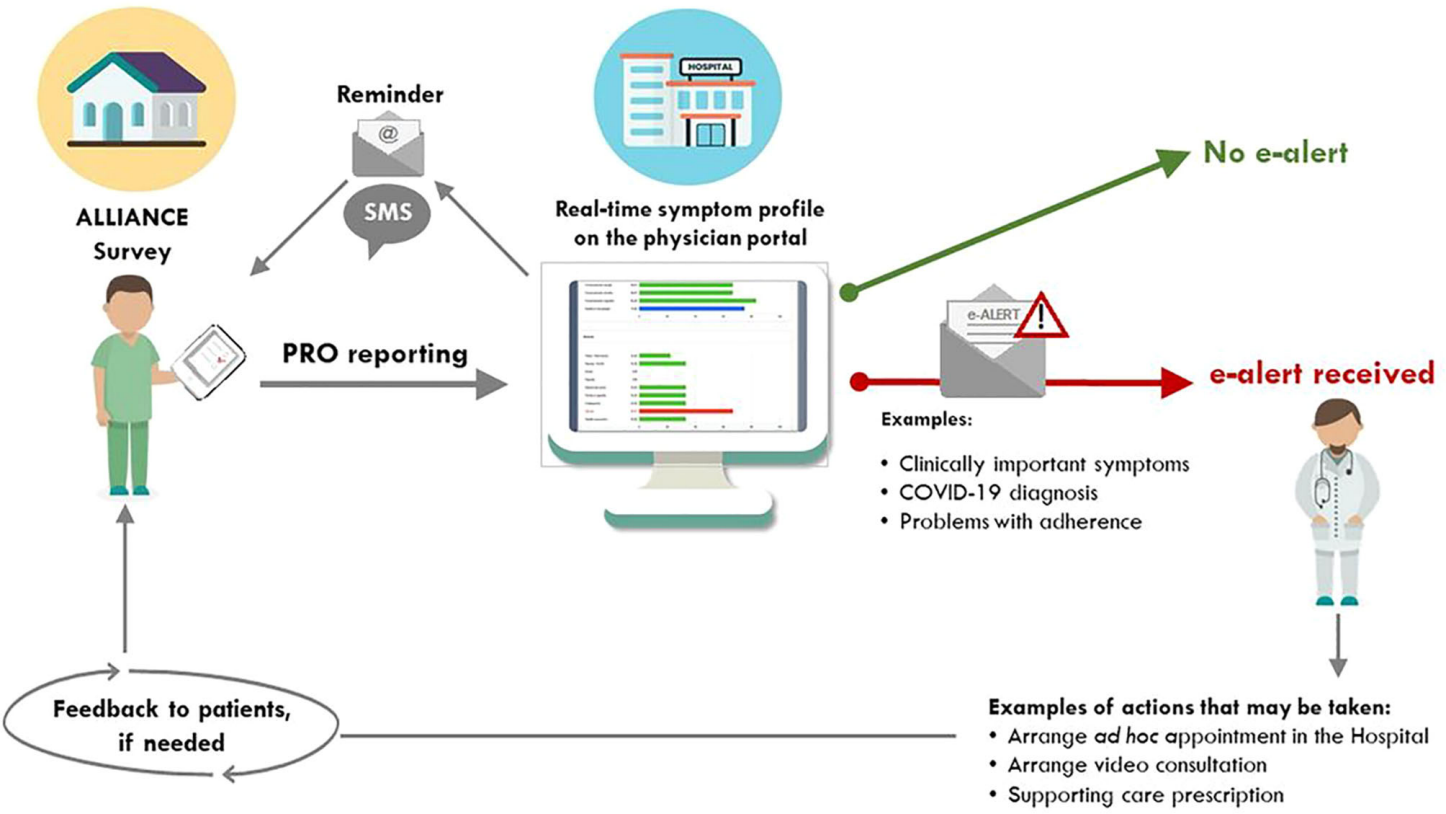
Schematic workflow of the patient-generated alerts to the medical team. PRO, patient-reported outcomes. This Figure was first published under the terms of the Creative Commons Attribution License (https://creativecommons.org/licenses/by/4.0/) in JMIR Research Protocols: Efficace F, Breccia M, Fazi P, Cottone F, Holzner B, Vignetti M. The GIMEMA-ALLIANCE Digital Health Platform for Patients With Hematologic Malignancies in the COVID-19 Pandemic and Postpandemic Era: Protocol for a Multicenter, Prospective, Observational Study JMIR Res Protoc 2021;10(6):e25271 (21). URL: https://www.researchprotocols.org/2021/6/e25271.

A brief schematic workflow of the data process is shown in **Figure 1**. After obtaining approval from the local ethics committee, and before being officially opened for recruitment, a start-up training session was organized by the GIMEMA-ALLIANCE management team. This session aimed to instruct the clinical staff of the participating hospital in using the platform and interpreting PRO data. SOPs developed for using the platform were illustrated during this online training session and also sent to the clinical staff just afterwards.

### Survey Evaluating Physicians’ Perception of Usability and Clinical Utility

For the purpose of this work, approximately after six months from the implementation of the platform, we asked the participating hematologists for structured feedback on their experience with its use, with a focus on their perception of its usability, clinical utility in their daily practice, and impact on quality of care. Only the hematologists who had registered at least one patient (also from the same center), were invited to complete the survey. Only one of the respondents was involved in the development process of the platform.

We developed an *ad hoc* web survey covering the following three broad domains: 1) usability and potential benefits; 2) monitoring of symptoms and health status; and 3) aspects related to physician-patient communication. Selection of items included in the Survey was based on consensus among the management Team and it was aimed at capturing the physicians’ perception of the specific features of the Platform.

The survey was implemented and administered online to physicians *via* REDCap (28). Each treating hematologist received a personal link through which they could enter and complete the online survey. Every two days, automatic reminders were sent to hematologists who had not yet completed the survey. Once the hematologists completed their survey, REDCap automatically saved the answers into a secure online database. Of note, REDCap was only used for the purpose of capturing physicians’ answers to the Survey and it had no role in the development or management of the GIMEMA-ALLIANCE Platform. The invited hematologists had two weeks to respond, and after this deadline, the survey was taken offline. The database with all the responses was closed and downloaded for statistical analyses. The characteristics of the enrolled patients and treating hematologists were summarized by proportions, mean, median, and range. Additionally, in order to check the possible association of the characteristics of hematologists with survey results, we performed a multivariable logistic regression analysis including the sex of the treating hematologists (male=1 vs. female=0) and the corresponding years of experience in dealing with hematologic patients as independent variables. The statistical tests we performed were bilateral, with α=0.05, set as the threshold for statistical significance. All analyses were performed using SAS software v.4 (SAS Institute Inc., Cary, NC, USA).

## Results

Between December 2020 and June 2021 (cut-off date for current analysis), 201 patients were invited to participate, and 180 (90%) accepted to enter the ALLIANCE platform. The median age of the patients was 57 years (range 21-91). The majority were diagnosed with chronic myeloid leukemia (n=32, 18%) or multiple myeloma (n=31, 17%). Overall, there were 89 (49%) of patients in stable disease. Twenty-three hematologists (44% males and 56% females) from 11 centers, with a median age of 42 years (range 31-63) and an average of 17 years (range 5-34) of experience in clinical practice completed the online survey.

### Usability and Potential Benefits of the Platform

All the treating hematologists agreed or strongly agreed that the platform was easy to use, and the majority agreed or strongly agreed (91.3%, n=21) that it is useful in the clinical management of their patients. Regardless of receiving the alerts when clinically important problems and symptoms occurred, 30.4% (n=7) of physicians entered the portal at least once a week to monitor their patients’ health status, while 30.4% did so at least once every two weeks. Only 21.7% (n=5) entered the portal less than once per month. The frequency of access on a regular basis was also independent of physician sex (p=0.393) and years of experience in clinical practice (p=0.404). After receiving the alert, the majority of physicians entered the portal the same day (60.9%, n=14) and made a phone call to their patients (69.6%, n=16). The hematologists often (30.4%, n=7) or very often (26.1%, n=6) used the ePRO information from the platform for their discussion with the patients, but this was not the case within their team. The same information was sometimes (30.4%, n=7), rarely (34.8%, n=8), or never (17.4%, n=4) used for discussion with other colleagues. Further details are presented in **Table 1**.

**Table 1:** Usability and benefits of the platform.

Item	Categories	n (%)
The automatic alert functionality is useful	*No*	1 (4.35)
	*Yes*	22 (95.65)
Most frequently undertaken action after receiving e-mail alert	*Phone call to the patient*	16 (69.57)
	*None*	5 (21.74)
	*Set up a visit in the hospital*	1 (4.35)
	*Other*	1 (4.35)
After how long the physician enter to the portal, after receiving the e-mail alert	*Within one day*	14 (60.87)
	*Within 2-7 days*	8 (34.78)
	*More than 15 days after*	1 (4.35)
Frequency of the access to the portal, regardless the e-mail alert receipt	*At least once a week*	7 (30.43)
	*At least once every two weeks*	7 (30.43)
	*At least once a month*	4 (17.39)
	*Less than once a month*	5 (21.74)
Use of the ePRO information from the platform for the discussion with the patients during clinical visits	*Very often*	6 (26.09)
	*Often*	7 (30.43)
	*Sometimes*	4 (17.39)
	*Rarely*	4 (17.39)
	*Never*	2 (8.7)
Use of the ePRO information from the platform for the discussion with the colleagues	*Very often*	2 (8.7)
	*Often*	2 (8.7)
	*Sometimes*	7 (30.43)
	*Rarely*	8 (34.78)
	*Never*	4 (17.39)
The platform is easy to use	*Strongly agree*	12 (52.17)
	*Agree*	11 (47.83)
	*Disagree*	0 (0.0)
	*Strongly disagree*	0 (0.0)
The platform is useful for the clinical management of the patients	*Strongly agree*	6 (26.09)
	*Agree*	15 (65.22)
	*Disagree*	2 (8.7)
	*Strongly disagree*	0 (0.0)

PRO, patient-reported outcomes.

### Monitoring of Patients’ Health Status and Symptoms Profile

Almost all the treating hematologists agreed or strongly agreed (95.6%, n=22) that the graphics about patients’ health status displayed on the platform were easy to understand and interpret. Sixteen physicians (69.6%) agreed and 3 (13.0%) strongly agreed that the platform helped them to better understand the patients’ general health status. Sixteen physicians (69.6%) agreed and 4 (17.4%) strongly agreed that the platform helped them to better understand the patients’ symptoms. Overall, 91.3% of physicians (n=21) agreed or strongly agreed that ePRO is useful to more accurately document patients’ symptomatic adverse events (AEs). In addition, 82.6% and 60.9% of physicians deemed ePRO information helpful to better identify low-grade and high-grade symptomatic adverse events, respectively. Further details are presented in **Table 2**.

**Table 2:** Evaluation of patients’ health status and symptoms profile.

Item	Strongly disagree n (%)	Disagree n (%)	Agree n (%)	Strongly agree n (%)
The graphics about patients’ health status are easy to understand and interpret	0 (0.0)	1 (4.35)	12 (52.17)	10 (43.48)
The platform was helpful to better understand patients’ general health status	0 (0.0)	4 (17.39)	16 (69.57)	3 (13.04)
The platform was helpful to better understand patients’ general symptom profile	0 (0.0)	3 (13.04)	16 (69.57)	4 (17.39)
The platform was used (at least once) for patients’ clinical management	1 (4.35)	3 (13.04)	17 (73.91)	2 (8.7)
ePRO useful to more accurately document patients’ symptomatic AEs	0 (0.0)	2 (8.7)	15 (65.22)	6 (26.09)
ePRO helpful to better identify low-grade symptomatic AEs	0 (0.0)	4 (17.39)	15 (65.22)	4 (17.39)
ePRO helpful to better identify high-grade symptomatic AEs	0 (0.0)	9 (39.13)	12 (52.17)	2 (8.7)

AE, adverse events; PRO, patient-reported outcomes.

### Physician-Patient Communication

Overall, 91.3% of physicians (n=21) deemed ePRO information useful to favor shared decision-making, and all of them considered this information helpful in suggesting supportive care strategies. Twenty hematologists (87.0%) deemed the information reported in the GIMEMA-ALLIANCE platform helpful in setting up unplanned visits with their patients and to enhance physician-patient communication. Only 13% of the treating physicians (n=3) did not agree with these statements. The details are presented in **Table 3**.

**Table 3:** Physician-patient communication and intention to use the Platform in the future.

Item	Strongly disagree n (%)	Disagree n (%)	Agree n (%)	Strongly agree n (%)
ePRO useful to favor share-decision making	0 (0.0)	2 (8.7)	17 (73.91)	4 (17.39)
ePRO helpful to suggest supportive care strategies	0 (0.0)	0 (0.0)	17 (73.91)	6 (26.09)
ePRO helpful to set up unplanned visits with the patients	0 (0.0)	3 (13.04)	18 (78.26)	2 (8.7)
ePROs useful to enhance physician-patient communication	0 (0.0)	3 (13.04)	14 (60.87)	6 (26.09)
Would use the platform also in the future	0 (0.0)	1 (4.35)	14 (60.87)	8 (34.78)
Would recommend the platform to other colleagues	0 (0.0)	2 (8.7)	10 (43.48)	11 (47.83)

PRO, patient-reported outcomes.

## Discussion

In this study, we explored the physicians’ perception of the usability and clinical utility of a digital health tool for ePRO monitoring in real-life hematology practice. While the clinical value of eHealth platforms has been well studied and documented in the context of solid tumors, less is known about their value in the context of hematologic malignancies.

Overall, our findings indicated a positive feedback from the hematologists interviewed, as most of them used the platform routinely, regardless of receiving automated alerts informing them about patients’ clinically relevant problems or symptoms. Additionally, graphically displayed ePRO results were found to be useful in enhancing patient-physician communication and in improving the detection of low-grade symptomatic AEs, by a large majority of respondents. This latter aspect may be of special relevance in routine practice across several hematologic cancer populations, such as those receiving long-term oral anticancer therapies. Indeed, it was previously observed that in these settings, patient-reported symptoms are typically of low to mild intensity and are therefore most likely to be unrecognized by the treating hematologist (5). Therefore, a better understanding of these chronic low-to-mild symptomatic AEs experienced by patients may have important clinical implications, for example, the adoption of more timely supportive care interventions. Results from the survey suggest that our platform may play a role in this respect, as all the physicians found it helpful in suggesting supportive care strategies. However, it should also be observed that there were 39% of physicians who did not find it useful to detect high-grade symptomatic AEs.

Recently, two studies evaluated the clinical utility and patient and staff feedback of ePRO systems (29, 30) in routine cancer care. In a non-randomized prospective cohort feasibility study, Kennedy et al. (29) explored the acceptability of an electronic system for collecting patient-self-reported AEs and quality of life. Staff feedback was positive, and 64% emphasized the benefits of receiving regular symptom reporting. In the PRO-TECT trial (30), 91% of the oncologists who responded to the survey found ePRO information useful, and this finding is consistent with that observed in our survey, where 87% of hematologists declared to have better understood patients’ symptoms by using the platform.

The clinical utility of ePRO systems is also linked to their ability to enhance patient-physician communication. In the PRO-TECT trial, 65% of the oncologists declared that they use PROs to often or sometimes guide discussions with patients (30), and this data is similar to our findings indicating that 74% of hematologists used (sometimes, often or very often) PRO information during clinical visits with their patients.

The active participation of clinicians is critical to enhance patients’ involvement and facilitate patient-centered care in routine practice. A recent study showed that the more clinicians looked at ePRO information from an online eHealth system (i.e., the eRAPID) before or during an appointment, the higher the patient engagement was with this system (13). One of the main challenges in implementing ePRO systems is clinicians’ reluctance to take on additional responsibility as well as perceived disruptions of the workflow (31). To minimize this risk, one solution may be to find physicians willing to engage their colleagues by demonstrating the flexibility of the tool, highlighting efficiencies in the overall work process, and convincing them of the value of the ePROs (31). It is also important to keep training physicians in the use of PROs with specialized training programs (32).

While we have documented a positive uptake of the use of this platform from the physicians’ standpoint, we cannot speculate on the patients’ perception of using this platform. However, a recent study that specifically examined the value of ePRO collection in the hematologic setting (including 102 patients with multiple myeloma and chronic lymphocytic leukemia) focused on the patients’ perception of the use of the portal and provided some reassuring data (19). The authors found that the majority of patients (84%) were willing to use the portal; however, they also observed that the completion of ePROs decreased over time, mainly because of the patient’s forgetfulness, and suggested ways to increase long-term participation rates (19). In another recent study, 227 lymphoma and chronic lymphocytic leukemia patients who completed web-based PRO questionnaires were randomized to care as usual (CAU), or to CAU plus return of PRO results (with or without a web-based self-management intervention) (20). No negative effects, for example in terms of psychological distress, were observed when individual PRO results were returned to patients, and authors concluded that this approach can be safely implemented in routine care practice (20).

The findings of our survey should be interpreted considering several limitations. It is possible that the positive results might be partly influenced by the characteristics of the sample, which consisted of physicians accepting to participate in the GIMEMA-ALLIANCE project. Hence, they are more likely to be enthusiastic about its use and reflect this positive perception in the rating of the survey. In addition, these findings should be regarded as preliminary, as the survey was performed approximately six months after the implementation of this tool and involved a small sample of hematologists. Additionally, our findings cannot be contextualized for a specific hematologic population or type of therapy. A key strength of our study is that it is one of very few reports documenting hematologists’ perception of the use of ePROs in real-life practice. In addition, we were able to document the feasibility of using the platform across several different institutions, each with different IT infrastructures and logistic support.

In conclusion, our results support the clinical utility, from the perspective of the treating hematologist, of integrating ePROs into the routine cancer care of patients with hematologic malignancies. Efforts are currently being made to put in place further educational and training activities for the use of PROs for hematologists involved and to implement novel IT functionalities that can further enhance its use in daily busy clinical practice.

## Data Availability Statement

The raw data supporting the conclusions of this article are available upon reasonable request to the corresponding author.

## Ethics Statement

The study was reviewed and approved by Comitato Etico dell’Università “Sapienza”. Also, each participating center obtained approval from its local ethics committee. The patients/participants provided their written informed consent to participate in this study.

## Author Contributions

FE and MV designed the study. FC and FE performed statistical analysis. FE and FS wrote the first draft. All the authors interpreted results and validated the manuscript’s content. All authors contributed to the article and approved the submitted version.

## Funding

This study was partly supported by the Associazione Italiana contro le leucemie linfomi e mieloma, Sezione di Roma (ROMAIL “Vanessa Verdecchia” Onlus) and by an unconditional contribution of AbbVie.

## Conflict of Interest

FE: Consultancy or Advisory Board: Amgen, AbbVie, Janssen, Takeda and Novartis. Research support (to his Institution) from AbbVie, Amgen and Novartis.

The remaining authors declare that the research was conducted in the absence of any commercial or financial relationships that could be construed as a potential conflict of interest.

## Publisher’s Note

All claims expressed in this article are solely those of the authors and do not necessarily represent those of their affiliated organizations, or those of the publisher, the editors and the reviewers. Any product that may be evaluated in this article, or claim that may be made by its manufacturer, is not guaranteed or endorsed by the publisher.
