# Supplementary material for: Physicians’ Perceptions of Clinical Utility of a Digital Health Tool for Electronic Patient-Reported Outcome Monitoring in Real-Life Hematology Practice. Evidence From the GIMEMA-ALLIANCE Platform
Source: Front Oncol. 2022 Mar 17;12:826040. doi: 10.3389/fonc.2022.826040 (PMC8968922; doi:10.3389/fonc.2022.826040)

Supplementary Figure 1. Example of graphical display of results of patient-reported functional aspects available on the physician portal. The red vertical bar indicates a clinically relevant problem in functional aspects.

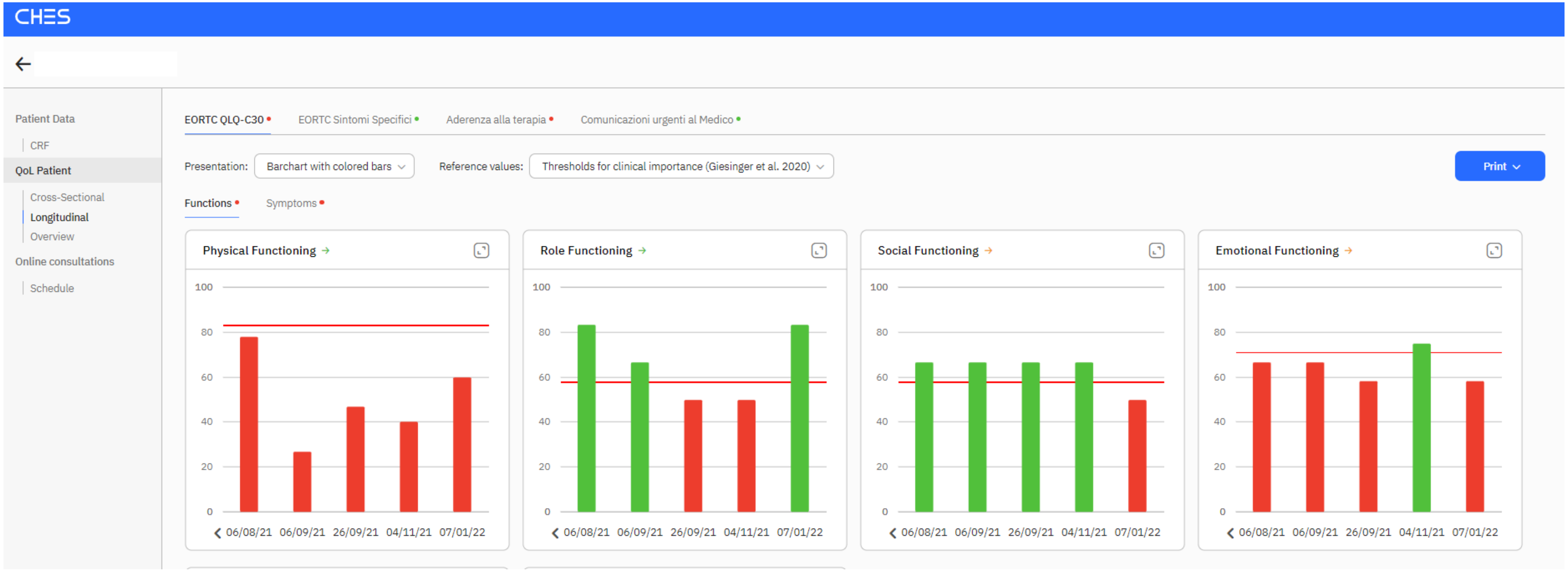

Supplementary Figure 2. Example of graphical display of results of patient-reported symptoms available on the physician portal. The red vertical bar indicates a clinically important symptom.

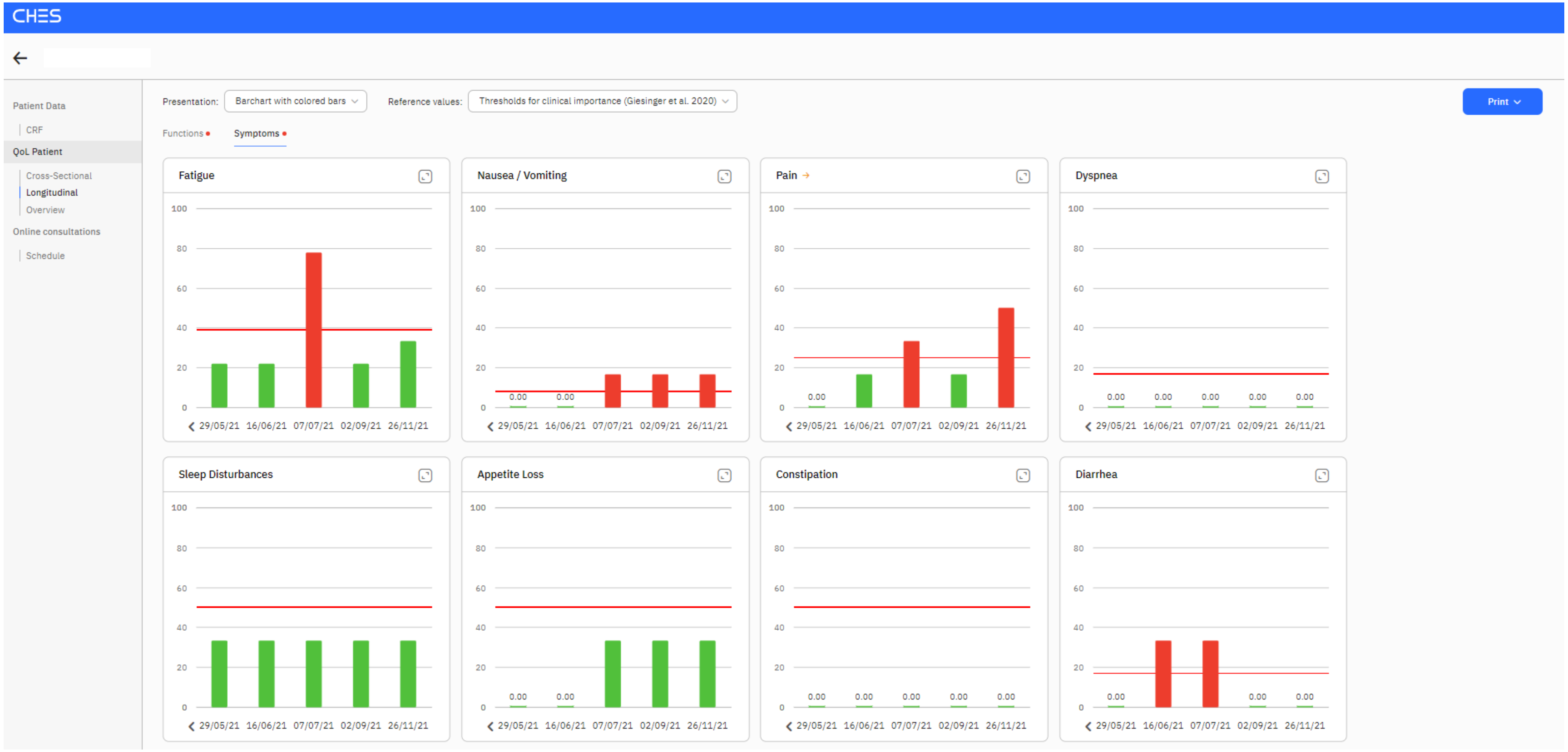

Supplement: Supplementary file 1 [file Image_1.pdf]
